# Re-thinking barriers to organizational change in public hospitals

**DOI:** 10.1186/s13584-017-0133-8

**Published:** 2017-03-20

**Authors:** Nigel Edwards, Richard B. Saltman

**Affiliations:** 1 Nuffield Trust, 59 New Cavendish Street, London, W1G 7LP UK; 2 Rollins School of Public Health, Emory University, 1518 Clifton Rd NE, Atlanta, GA 30322 USA

**Keywords:** Health system governance, Governing public hospitals, Hospital management, Health policy, Organizational behavior

## Abstract

Public hospitals are well known to be difficult to reform. This paper provides a comprehensive six-part analytic framework that can help policymakers and managers better shape their organizational and institutional behavior.

The paper first describes three separate structural characteristics which, together, inhibit effective problem description and policy design for public hospitals. These three structural constraints are i) the dysfunctional characteristics found in most organizations, ii) the particular dysfunctions of professional health sector organizations, and iii) the additional dysfunctional dimensions of politically managed organizations. While the problems in each of these three dimensions of public hospital organization are well-known, and the first two dimensions clearly affect private as well as publicly run hospitals, insufficient attention has been paid to the combined impact of all three factors in making public hospitals particularly difficult to manage and steer.

Further, these three structural dimensions interact in an institutional environment defined by three restrictive context limitations, again two of which also affect private hospitals but all three of which compound the management dilemmas in public hospitals. The first contextual limitation is the inherent complexity of delivering high quality, safe, and affordable modern inpatient care in a hospital setting. The second contextual limitation is a set of specific market failures in public hospitals, which limit the scope of the standard financial incentives and reform measures. The third and last contextual limitation is the unique problem of generalized and localized anxiety*,* which accompanies the delivery of medical services, and which suffuses decision-making on the part of patients, medical staff, hospital management, and political actors alike.

This combination of six institutional characteristics – three structural dimensions and three contextual dimensions – can help explain why public hospitals are different in character from other parts of the public sector, and the scale of the challenge they present to political decision-makers.

## Background

### Framing the question

Hospitals are hard organizations to change. Publicly owned and operated hospitals even more so. In many countries, both managers and staff anticipate, despite constant calls for improvements in efficiency, quality, and responsiveness, that little will be different tomorrow or next year. Similarly, politicians, seeking to outflank these intra-institutional expectations, have learned to introduce major reforms early in their term to maximize what leverage they have over public hospital managers and staff.

This creates a narrow window for organizational change in public hospitals which, combined with the standard political hazards [1], still has only a relatively low probability of success, and thus further reinforces the view of hospital management and staff that ‘this too shall pass’.

Conversely, while public hospitals remain broadly insulated from major policy-driven change, there continues to be rapid successful change in the practice of medicine. This can be seen in clinical treatments, diagnostic technology, in reduced lengths of stay and increased ambulatory treatment, all in spite of a shift to patients being older, more complex and with increasing amounts of chronic disease.

Taken together, these conflicting trends result in most public hospital change being technical and functional rather than organizational and institutional. Even as clinical responses change relatively rapidly, departmental and institutional routines remain broadly insulated, ensuring organizational stability for many regular daily activities. This can be an important positive from the perspective of hospital staff, and, sometimes, patients, but not always for the latter if it means long waits in a traditional outpatient department or the other tedious things that patients have to do to accommodate ingrained hospital routines. In routine operating aspects, stability also can mean organizational stasis and inertia regarding clinical issues as well as larger policy and management objectives – an important negative from the perspective of health sector reform [2].

This intricate pattern of public hospitals’ internal resilience and external resistance can be observed in a variety of countries and in a range of different financial and institutional circumstances. Among the most visible examples have been the mixed success achieved over the past 20 years to eliminate unacceptably long waiting times for elective procedures in English and Swedish hospitals [3, 4]. Further examples can be observed in the slow pace of organizational and institutional change in public hospitals following major reform in the English NHS [5] and resistance to structural change in the Norwegian [6] health systems; the unsteady development of hospital level semi-autonomy in some newly built hospitals in some Spanish provinces [7], and the complexity of introducing elements of institutional self-management in Estonia [8]. Examples of similar reactions to efforts to introduce major organizational change can be found in Portugal [9] as well as in Israel [10].

Academics have proposed a range of theories to explain and/or mitigate this common public hospital behavior. In the early 1990s, there was discussion among political scientists about path dependency as the source of institutional obduracy [11, 12] as well as the potential value of “big bang” as against “incrementalist” approaches to generate change [13]. The early 1990s also saw the rise of New Public Management theory, calling for private-sector-derived managerial strategies such as contracting out services [14] and “steering not rowing” in the organization of public sector service provision [15]. Public Administration theorists explored why contracting in public sector institutions, once introduced, was difficult to manage effectively [16]. More recently, ideas from complexity theory have been invoked [17], although these perhaps have more utility in explaining why organizational change is so difficult than they do in identifying strategies that might achieve it.

Inside the health care sector, a variety of fiscal and regulatory solutions have been put forward to try to address this conundrum. Paying hospitals according to Diagnostic-Related Groups (DRGs) changed the financial incentives for hospital managers [18], and, in some cases, clinicians. Attempts were made to increase the power of these incentives by adding pay-for-performance to DRGs to target hospital staff behavior more effectively [18, 19] so as to reflect different value streams within healthcare [20].

Throughout this period, turning away from public sector control by creating semi-autonomous public hospital management [21], private sector contract management for public hospitals [15, 22], privately built and managed but publicly paid new public hospitals [23, 24], and also full privatization of existing public hospitals [25, 26] have all been suggested and, in a number of countries in different circumstances and with a wide range of limitations, introduced. Simultaneously, in tax-funded health systems like England, a panoply of new regulatory bodies were established (National Institute for Clinical Excellence (NICE); Commission on Health Improvement (CHI); Monitor; Care Quality Commission; etc.) to try to rein in poor quality and/or inefficient managerial practices in individual institutions.

All of these measures have had at least some effect on hospital behavior and institutional outcomes [27, 28]. This has been generally positive but the effect has often been less powerful than expected and not necessarily what was intended [29]. However, most evoked reaction from the forces of institutional status quo, and nearly all have had a relatively short half-life in generating effective organizational change [30].

This complex response from public hospitals is not always inappropriate or misguided [31]. Many of the characteristics of successful organizations and professional and managerial practice can easily tip over into behaviors that thwart change. This poses questions which are a core governance issue for both policy and management in public hospitals: when does the positive and necessary need for day-to-day organizational stability decay into a negative and obstructive form of organizational stasis?

- Where is the boundary between organizational resilience and organizational resistance?
- When do appropriate professional interests and caution evolve into inappropriate resistance to change?
- When does organizational or professional culture cross the line into territorialism?
- When does the hospital’s concern about income stability become protectionism?
- When does top-down regulation become unhelpful interference in essential managerial prerogatives?

Who makes these decisions, and what type of response is possible? In privately owned hospitals, responsibility rests with senior management, and their jobs may well depend on how rapidly and effectively they respond. But how does this major institutional process play out in public hospitals?

### This paper’s approach

This article sets out a framework for conceptualizing why public sector hospitals are so hard to successfully change and suggests a pathway toward better strategies to address these issues. It makes two tightly intertwined arguments.

The article’s first contention is that, to date, there has often been poor specification of the problem. In particular, the traditional metaphors or analytic frameworks used to steer decision-making for public hospitals continue to be conceptually limited and insufficient for thinking about the problems they are supposed to frame.

The second argument examines the core structural problems that public hospitals present. The paper contends that there are three separate structural characteristics which, together, inhibit effective problem description and policy design for public hospitals. These three structural constraints are i) the dysfunctional characteristics found in most organizations, ii) the particular dysfunctions of professional health sector organizations, and iii) the additional dysfunctional dimensions of politically managed organizations.

While the problems in each of these three dimensions of public hospital organization are well-known, and the first two dimensions clearly affect private as well as publicly run hospitals, insufficient attention has been paid to the combined impact of all three factors in making public hospitals particularly difficult to manage and steer.

Further, these three structural dimensions come together in an institutional environment shaped by a set of three external contextual factors that further constrain effective management and reform of public hospitals.

The first contextual limitation is the inherent and increasing complexity of delivering high quality, safe, and affordable modern inpatient care in a hospital setting and across organizational boundaries [32].

The second contextual limitation is a set of specific market failures in public hospitals, which limit the scope of the standard financial incentives and reform measures. These mechanisms are also often in conflict with the role of professional medical authority (which has been already discussed above as the second structural limitation in hospitals).

The third and last contextual limitation is the unique problem of generalized and localized anxiety*,* which accompanies the delivery of medical services, and which suffuses decision-making on the part of patients, medical staff, hospital management, and political actors alike.

Thus the paper’s second argument is that this combination of six institutional characteristics – three structural dimensions and three contextual dimensions – helps explain why hospitals generally, and public hospitals in particular, are different in character from other areas in the public sector, and the difference in the demands they place on political decision-makers. It also suggests the importance – indeed the primacy – of sophisticated organizational level management as the key building block of effective public hospital behavior, and the consequent importance of correctly aligning policy to facilitate that core central objective.

The central argument of this paper can be summarized, then, as follows. First, existing standalone analytic frameworks inadequately describe the policy and management quandary that public hospitals present. Second, the three plus three analysis delineated above provides a better (but probably still incomplete) framework for understanding and re-directing public hospital decision-making and outcomes. The sections that follow below explore these ideas in more detail.

## Main text

### Partial and inadequate analytic frameworks

A central issue in the reform of hospital systems is a poor specification of the problem and consequently of the methods set forward for dealing with that problem. One reason for this difficulty seems to be the complexity of these systems, which makes the identification of root causes difficult and creates many feedback systems which render the outcomes of policy interventions unpredictable. An understandable approach to complexity is to look for ways of simplifying it and in these circumstances solutions based on a particular narrative, ideological position or method look very attractive. The difficulty is how to make the problems manageable while not losing core elements of the complex reality, which are essential for appropriate diagnosis and solution.

Firstly, there are issues about the understanding of the system that is being reformed [23]. There is often a focus on hospitals which confuses institutions or buildings with the actual operating and business models that underpin them, and which fails to grasp the interconnections between the component parts of hospitals and the wider health system.

Secondly, there is the conceptual lens through which problems are defined. There are several in use and some are used together:

- *Economics and markets*: The problem of poor performance is due to the absence of appropriate financial incentives and the effective operation of market mechanisms such as competition, the threat of bankruptcy and new entrants at the individual and institutional level.
- *Business models*: Michael Porter argues that health care has the wrong sort of competition [33]. Similarly, Clayton Christensen argues that current business and operating models do not fit the nature of the work to be done [34].
- *Theory X Management*: The problem is seen to be the result of a lack of firm managerial grip and control. This leads to the use of performance management measures with heavy emphasis on sanctions for poor performance. Finance ministries often seem to be attached to this model [35].
- *New Public Management*: Measures to contract out clinical and non-clinical services to both public and private providers, combined with public-private-partnerships to build new facilities, will generate better performance [14]. Patient choice of public or private providers, with money following patients, will reinforce pressure for higher quality outcomes [36, 37].
- *Complex Adaptive System*s: Health care should be seen as non-linear and dynamic, composed of independent agents with conflicting goals and intelligent adaptive behavior, resulting in self-organization [38, 39]. This model is more comprehensive, but is less helpful as a guide to specific policy or management action.

Moreover, all of these analytic frameworks may have different (usually implicit) assumptions about the nature of human behavior and motivation. For example, readings of how far policymakers view staff and managers as ‘knights’ or ‘knaves,’ to use Le Grand’s typology [40]. In other words, the extent to which it is possible to rely on intrinsic motivation, professionalism and good intentions (‘knightly’ behaviors) rather than having to use a variety of incentives, sanctions, inspection and other methods to control self-interest and less noble motives.

### Three structural sources of public hospital resistance to change

Each of the three structural sources of dysfunction has its own internal metaphors, incentives and constraints. While each source suggests a clear reform roadmap itself, the different approaches interfere with each other, and no one approach covers all three sources of obstruction. Hence to date the reform roadmaps put forward for public hospitals to deal with organizational dysfunction have been necessarily partial and insufficient.

#### Normal organizational stasis and dysfunction

The resistance of large scale organizations to managerial systematization has been analyzed and debated in organizational sociology and public administration for a hundred years. At the turn of the 20th century, the German sociologist Max Weber posited that all large organizations were necessarily “legal-rational” bureaucracies, organized through a system of rules and offices:

“The choice is only between bureaucracy and dilettantism in the field of administration” [41].

“Only by reversion in every field – political, religious, economic etc. – to small-scale organization would it be possible to any considerable extent to escape its influence” [41].

In 1911, a mechanical engineer named Frederick Taylor published an account of what he termed “scientific management”, in which he argued that work inside organizations could be broken down into small tasks, measured, controlled and optimized by managers [42]. Two decades later, trying to validate Taylor’s thesis, the Harvard sociologist Elton Mayo founded the field of organization theory with his 1930s studies of work processes in the Northern Electric Company’s Hawthorne plant. Mayo discovered that the very process of measuring a worker’s performance improved it – the so-called “Hawthorne Effect” – making it possible to begin to break down the “systematic soldiering” by which assembly line workers set production rates and maintained control over manufacturing output [43]. Subsequent organization theorists sought to develop new strategies to counter regular organization-wide resistance to new managerial methods and techniques. Among other insights, they proposed using salary levels to create “a zone of indifference” within which employees would accept direction [44]; argued that decision-makers inside organizations were restricted by “limits of rationality” to only a few practical choices [45]; and suggested that managers who approached workers as intelligent team members (“Theory Y”) achieved more than those who treated their workers as indifferent obstacles (“Theory X”) [46]. Proposals also were made to treat organizations as cybernetic feedback-based systems [47].

Quite differently, sociologists and anthropologists attributed organizational stasis to deeply ingrained characteristics of the workers inside large formal organizations. The French sociologist Michel Crozier’s research detailed how publicly operated organizations become trapped in a vicious circle of underperformance where permanent strategies generated by employee groups to increase their own discretion provoked overly tight and counter-productive managerial controls [48]. Geert Hofstede, a Dutch corporate anthropologist who did much of his research on IBM, found that efforts by senior management to change organizational culture – how employees did their work – slowly but inexorably were eroded back to the original organizational norms and values:

“Institutions may be changed, but this does not necessarily affect the societal norms, and when these remain unchanged, the persistent influence of a majority value system patiently smooths the new institutions until their structure and functioning is again adapted to the societal norms” [49].

Taken together, these theories add up to a clear if somewhat inchoate set of explanations for why normally functioning organizations – including both public and privately operated hospitals – present structural barriers to efficient functioning and introduce seemingly inexorable perverse consequences.

A number of these organization theories have been applied to explain the structural resistance to change specifically within public hospitals. Saltman found that physicians and nurses in Danish and Dutch public hospitals had “permanent group strategies” that substantially constrained both managerial and political initiatives for institutional development [50, 51]. Saltman and Bergman [52] and Saltman [4] suggested that core Swedish societal norms and values provided both stability and stasis regarding change in the Swedish health care system.

#### Health sector/Professional stasis

Beyond the standard “normal” level of dysfunction that organizational theorists attribute to all large organizations, specifically health care institutions contain a further, second structural impediment to their positive organizational development. This second source of resistance to change reflects the role, authority and institutional prerogatives of the health care professionals that are the core employees within it. The inherent complexity of managing health professionals – and particularly physicians – has been studied by medical sociologists and management strategists at least since the middle of the last century [53–55]. The concept of a “disconnected hierarchy” [56] has been effectively used to characterize the difficulty of hospital managers, who lack the means to control important positive reinforcers for physicians (eg peer respect, publication in peer-reviewed journals) and thus must rely on less effective, negative actions such as threats of reduced funding or space [57]. While subsequent research has become more targeted, the central imbalance in decision-making authority that particularly physicians introduce into health care organizations generally, and hospitals in particular, remains unresolved.

On the clinical side, managers have sought to develop strategies that harness physicians to medical teams as a way to create better congruence between physician decisions and the best interests of the hospital they work in [58, 59]. As clinical data on individual performance has become more available, research has sought to determine the most effective way to improve physician performance. However, these internal management strategies have had relatively short half-lives, even shorter if the physician’s medical practice and salary are not tightly tied to the financial position of the hospital (either because the physician is in private practice on contract to a public hospital – as is often the case in The Netherlands – or if the physician is a public employee in a permanent post and thus insulated from most management rebuffs) [60–62]. A key finding from research in this field is that physicians tend to dominate hospital decision-making procedures and constrain undesired institutional policies and practices regardless of the national health system and culture they operate within [50, 63–65]. Operationally, physicians largely control the rate and pace of their workloads and the workflow of the rest of the organization, typically prevailing in conflicts with other staff groups [66].

As the prior paragraph suggests, the macro and micro political power of the medical profession to exert influence is considerable and well documented. Their trusted position in society, control over how resources are used, their monopoly of special knowledge and other sources of power, deference, and influence are very significant in both creating and slowing institutional as well as organizational change.

From the patient’s perspective, strong physician influence is often seen as a good thing: the last thing a sick patient wants is for a clinical decision to be influenced by or, worst of all, made by non-medically qualified administrators or lower-level medical staff. This patient support reinforces the particular physician-led character of decision-making inside hospitals, and with it the inevitable resistance to externally generated change – be it politically or managerially led – that could interfere with physician and, more broadly, medical staff led decision-making.

#### Governance induced/Political stasis

The third area of organizational dysfunction that public hospitals suffer from reflects the explicit political character of policy and management decision-making in these institutions. There is no shortage of literature that describes the non-linear, non-optimizing, and sometimes seemingly non-rational elements that compose typical politically structured decision-making in all sectors of public policy (see for example [67, 68]). To suggest that public sector decision-making is broadly dysfunctional when viewed from the perspective of health provider and/or service organizations as well as of the staff working in those institutions is not novel.

Critiques of political decision-making focus on two inherent operational problems: legislative roadblocks and administrative/implementation rigidities. Political scientists have written at length about the problems of writing and passing effective legislation: the importance of “policy champions” and “windows of opportunity” as well as the always-present dangers involved in building coalitions and evading veto-points [1]. The German Chancellor Bismarck, somewhat less eruditely, is credited – perhaps incorrectly^1^ – with quipping that “those who love sausage and the law should never watch either being made”.

Administrative and implementation difficulties also have received considerable scholarly attention. Drawing on the understanding of public organizations put forward by Weber [41] and Lindblom [69], among many others, sociologists and political scientists have detailed the procedural difficulty that public agencies have in transforming legislated policy into effective practice. Lipsky showed how front line staffers became “street level bureaucrats” who de facto re-wrote official rules in welfare service agencies so as to obtain higher benefit levels for their clients [70]. Concepts such as “bureaucratic capture” by outside interest groups and “bureaucratic cooptation” through briefs and submissions from interested parties also demonstrate the bending of administrative decision-making to satisfy political needs. Going one step further, Pressman and Wildavsky highlighted how hard it is to get multiple government agencies to cooperate effectively in the implementation of public sector projects even when there is policy agreement among their respective agency heads [71]. Hood suggested that adoption of private sector tools such as contracting out were essential to overcome these various types of politicized administration and to establish properly accountable service delivery [14]. Osborne and Gaebler summarized these different implementation perspectives into a generalized need for public sector governmental officials to “steer more and row less” [15].

Further, on an operational level, administrative behavior in a public civil servant environment also involves a number of rigidities that one could delicately summarize by the term “bureaucratic”. Staff often have legislatively protected jobs and pensions, with little incentive to take risks or be innovative. Efforts to sanction or dismiss public employees, particularly when they are unionized, are complex, elongated, expensive, and as an unsurprising consequence, rarely pursued. Whatever one might think about the appropriateness of such job security for postal or pension office workers, the same civil servant status creates unique challenges in the decidedly different working environment of emergency and inpatient medical care services. Conversely, some public systems also have the opposite problem. The political appointment of hospital managers creates instability, can lead to the promotion of under-qualified candidates and leads to short termism and the avoidance of contentious issues.

Beyond these structural problems inherent in legislation and administration, there are a number of specifically health sector dilemmas that require difficult policy decisions which are never permanently resolved. The superiority of decentralized as against centralized and/or re-centralized models of decision-making is continually debated, with different variants of Rondinelli’s four forms of decentralization (devolution, delegation, deconcentration, privatization) taking turns as the favored arrangement [72]. Present-day proposals in Norway to centralize nationally the ownership and supervision of all hospitals, and in England and Finland to combine health and social services within a single administrative structure, suggest that currently the perceived superiority of centralization is seeing a revival in a number of tax-funded European countries [73].

Political decision-makers similarly have found themselves boxed in by the multiple conflicting rationalities that apply to making policy for health provider institutions. Among the most prominent are:

- the conflict for resources between applying the rule of rescue (expanding clinical and emergency facilities) as against the need to expand population-based equity (prevention activities and population/geographic dispersal of core services)
- the conflict between expanding curative and primary care coverage areas as against staying within financial and budgetary limitations
- the conflict between ensuring long-term fiscal sustainability as against getting re-elected
- the conflict between satisfying staff and/or union demands as against satisfying patient needs.

An area of policy decision-making that never goes away is the question of the public/private mix in service provision. While a number of European countries now view this issue as a pragmatic one tied to care standards and performance [74], it is still a live political issue [75, 76].

All these policy conflicts reduce the flexibility available to key health sector decision-makers, and lend credence to those who prefer to “not rock the boat” thereby favoring stability and/or stasis. Faced with these uncomfortable and sometimes medically uncertain concerns, many political actors prefer to find less major, less threatening policy issues to tackle which may be popular but are more peripheral and less likely to evoke opposition.

### Three contextual sources of public hospital resistance to change

Each of the three contextual factors that constrain organizational change in public hospitals reflects the interaction of key elements in the external environment with essential internal characteristics of mission, focus and efficiency. While two of these factors – complexity and anxiety – affect private as well as public hospitals, the third, market failures, has particular impact on efforts to introduce and sustain effective financial and management incentives specifically on publicly owned and operated institutions.

#### The complexity of organizing high quality, effective health care

The extraordinary capacity of contemporary medicine to intervene and/or restore human functioning requires high levels of technically sophisticated and financial costly capital equipment, carefully configured physical space, and high levels of coordination and cooperation among different categories of medical and non-medical staff both within and beyond hospitals walls. These institutional requirements to deliver good medical care take great organizational focus and managerial skill to achieve and sustain. The continued expansion of diagnostics and treatments, and the associated safety hazards, has added a further dimension to hospital complexity.

There are also large and growing regulatory requirements that governments place on hospitals – extending from normal requirements of labor law and handling dangerous materials (eg radioactive waste) to specialized concerns about care quality and patient rights [29, 77]. For large public hospitals with extensive acute care facilities, all these factors add incrementally but also, taken together, fundamentally to the major managerial challenge of running an effective, efficient and safe institution.

#### Market failure in public hospitals

European health systems have struggled to define the appropriate role of market mechanisms in hospital management for 30 years. Suggestions were initially made in the mid-1980s that competitive public sector budgeting could create an “internal market” [78] by transforming public hospitals into “public firms” that could compete with each other for patients [36], and various concepts such as “planned markets” and “quasi-markets” [36, 37] received considerable attention from various national policymakers. Similarly, the development of New Public Management thinking in the area of Public Administration reinforced the logic of applying market mechanisms in order to better achieve public sector fiscal and managerial outcomes [14].

National policymakers have often had difficulty finding the balance between generating effective market pressure, their desire for control and maintaining core public health care obligations and responsibilities. This has been seen in the reluctance of political decision-makers to let semi-autonomous hospital managers retain decision-making leeway, and the frequency with which political officials clawed back control [21]. This is playing out again with recent reassertion of central control in the United Kingdom, Hungary and Norway. Hospital trusts, for example, have gone through a variety of different structural formats and configurations in England, Spain, Norway and Italy.

Efforts to develop market-sensitive managerial arrangements for public hospitals based on volume-sensitive contracts have often been re-designed and/or de-emphasized due to concerns about inappropriate incentives regarding quality and access in tax-funded systems like Stockholm County, Sweden, and in a number of different English trusts.

Similarly, the relatively few efforts to contract out public hospital management to private (typically for-profit) companies have proven contentious, with little agreement as to whether outcomes have been improved in such examples as Alzira in Valencia, Spain and Sct Gorans in Stockholm, Sweden [79, 80]. This overall difficulty of fit has further inflamed broader policy disputes over the appropriateness of market mechanisms – or new public management strategies such as contracting out to private sector entities for specific services – having any role in public hospital management [76, 81].

One particular dilemma is that the cost structure of hospitals and the interconnectedness of services makes the reduction of costs when income is lost very challenging. This means that the preferred strategy for hospital managers is to grow the organization and to compensate for a loss of income from one source with increased volumes from existing payers or new work from other payers rather than reducing costs. This, combined with the reluctance of politicians to let public hospitals go bankrupt, creates a further market failure.

Although patient organizations have recently become more vocal inside countries and across the European Union, demanding a greater role in political and policy decisions that affect patient outcomes [3], the voice of the consumer in many public hospital systems is relatively weak and there is not always choice of provider further limiting the effectiveness of market mechanisms.

#### Generalized and specific anxiety

The reasons for patients to be anxious are obvious and real, and need not be recounted here. Staff experience anxiety as well from the emotional and physical labor involved in caring, in breaking bad news, making difficult decisions and having a large number of opportunities to make errors which may result in death or disability [82, 83]. Patients and clinicians are confronted with existential issues that have challenging psychological resonances. Clinical detachment and other coping strategies are often of limited effectiveness and can compound the problem. Exhortations to be compassionate may make the problem still worse. This tends to be compounded by an environment that is hierarchical and quick to attribute blame: these may in fact be defenses against anxiety but they often have the paradoxical effect of increasing it [83, 84].

Managers have the anxiety that comes from the limited control that they can wield in a professional organization, often combined with high levels of external pressure to perform and a political context that tends to denigrate their role and limit their scope of action. In turn, politicians are frequently in the same position and are held accountable for a system they cannot control and which they will not be associated with for long enough to change. The fact that they may not even have formal responsibility for the way that care is delivered is not a protection from blame. The experience of trying to reform health systems and the subsequent career trajectories of those that do suggests that their anxiety may be justified. Both political and managerial anxiety seep into the clinical system as attempts are made to create assurance and exercise control.

Unresolved anxiety in organizations can lead to a range of dysfunctional behaviors including bullying, depersonalisation, ritualized behavior, techniques for blame shifting or diffusion, approaches that reduce the chance to learn from failures and – crucially for this paper – resistance to change.

### Summing the public hospital quandary

**Fig. 1:**
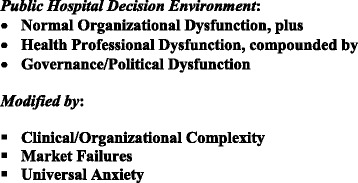
The 3 + 3 decision framework

Taken together, the three structural plus the three contextual factors described above steer public hospital decision-making toward both organizational and institutional resistance to change. The fundamental structural bias is toward not doing rather than doing, toward not implementing rather than implementing, and toward minor rather than innovative and systematic change. Moreover, while the effect of these six constraints varies across countries, across governments, and also within different areas of countries, the overall consequences are apparent quite widely. Simply put, the process of organizational change in public hospitals faces a highly resistant institutional environment, captured by the formulation in Fig. 1.

Public Hospital Decision Environment:

- Normal Organizational Dysfunction, plus
- Health Professional Dysfunction, compounded by
- Governance/Political Dysfunction

Modified by:

- Clinical/Organizational Complexity
- Market Failures
- Universal Anxiety

This set of structural and contextual factors breaks out the core organizational constraints that confront decisions about change in public hospitals. These six dimensions delimit the de facto boundaries, lay down the practical parameters that both policymaking and management needs somehow to accommodate. Every proposal to alter institutional behavior, every new mechanism to improve quality and safety, to introduce more efficient financial programs, to alter daily staff service routines, even to change contracting partners for auxiliary services such as laundry or cleaning, all necessarily gets filtered through this six-way sieve before the outcome actually emerges at clinic or organization level.

This complicated and complicating mix of factors simultaneously generates the need, on the one hand, for continuous institutional development and change, and, on the other hand, reinforces the multiple organizational, professional, political and behavioral forces pushing for stability and/or stasis in day-to-day organizational operations and procedures.

As with all change, understanding behavior and motivation are important. This framework needs to be informed by a richer and more nuanced view of this than is the case in some of ideas that have underpinned policymaking. The framework does not solve the problem of how to get complex groups of individuals aligned behind change objectives, it can help understand some of the issues that make this difficult.

## Conclusions

Taken overall, the above framework of structural limitations and contextual factors can help health sector analysts better understand how public hospital decision-making actually takes place. Specifically, the 3 + 3 framework outlined in Fig. 1 can serve as an intellectual sieve through which to test the feasibility of potential reform ideas. By exploring how well a possible policy or management strategy might work in dealing with dilemmas raised in each of the framework’s six separate dimensions, analysts potentially can adjust or re-frame one or more elements of a particular approach, seeking to ensure that it would improve, or at least not make worse, an existing element of the overall public hospital decision-making equation. This approach would allow new reform strategies to in effect be “stress-tested” across the six different dimensions that define the public hospital decision environment. Moreover, policy and regulatory initiatives can be assessed for their explicitly managerial impacts. This modelling of different likely institutional, organizational, and contextual responses can likely reduce practical and/or operational difficulties within one or more of the six different decision-related factors prior to a new strategy being introduced into practice.

Building out a set of useful and implementable responses to this analysis will require a carefully choreographed mix of regulatory and managerial activities, particularly in the current difficult environment for public hospitals generally [85]. As Dixon-Woods [86] noted earlier of quality programs, there is a danger that too much technical complexity can overwhelm the ability to successfully implement reforms in practice that on paper had appeared to be effective interventions. Dixon-Woods’ antidote of practical wisdom and small politics, the notion of “humble inquiry” at the center of Edgar Schein’s recent work [87], and also the strategy at successful medical centers like Virginia Mason whose Chief Executive Officer has called for “authentic relationships built on trust and respect” offers some answers.^2^ There may be little alternative to hard work, the use of data, attention to detail, the deployment of a method and a clear vision set from above [88].

The 3 + 3 framework can be of particular value to national ministries of health, where the importance of gaming out the inter-relationships between political and/or regulatory initiatives on the one hand, and likely managerial responses at the organizational level on the other, is paramount to the long-term success of health sector reform initiatives. Fine-tuning new programs and strategies to accommodate the 3 + 3 framework also could help achieve broader national priorities of which more effective management of public hospitals forms an important component.

It is important to note that effective design principles necessarily start from the premise that the organizational problems created by the three dominant dimensions of dysfunctionality, as well as the behavioral problem of generalized anxiety, cannot be solved by clever new policy proposals devised at the political level. They will only yield – and only to some extent – to re-thinking how to engage the people inside the organization who receive operational directives, and the structural environment and practical management realities on the ground that determine how they respond to those directives.

It also is important to recognize that the 3 + 3 framework is not a formula for discovering what organizational theorists sometimes derisively refer to as “magic bullets”. Instead it is a call to apply the basic core lessons of traditional hands-on, shop-floor-level management. Among other elements, this approach will mean accepting different management styles and approaches in different institutions, indeed in different parts of the same institution. Operational flexibility, not centralized standardization, will need to become the policy watchword. Importantly, the political role in public hospitals will need to fade further, while the managerial role will necessarily grow in prominence.

How such an approach could be conceived, designed, implemented and sustained in day-to-day real-world practice raises a raft of difficult questions. A wide-ranging discussion among all key constituencies will be necessary to begin to detail the complex process of change that will be required.

## Abbreviations

DRGs: Diagnostic-Related Groups

## Footnotes

1 The Daily Cleveland Herald, March 29, 1869, quoted lawyer-poet John Godfrey Saxe as saying that, “Laws, like sausages, cease to inspire respect in proportion as we know how they are made”, and this may be the true origin of the saying.

2 GS Kaplan, 2013, comment on back cover of EH Schein (2013).
